# A social network analysis of the spread of COVID-19 in South Korea and policy implications

**DOI:** 10.1038/s41598-021-87837-0

**Published:** 2021-04-21

**Authors:** Wonkwang Jo, Dukjin Chang, Myoungsoon You, Ghi-Hoon Ghim

**Affiliations:** 1 Department of Public Health Sciences, Graduate School of Public Health, Seoul National University, Seoul, Republic of Korea; 2 Department of Sociology, Seoul National University, Seoul, Republic of Korea; 3 The Institute for Social Data Science, Pohang University of Science and Technology, Pohang, Republic of Korea; 4 CYRAM Inc., Seongnam, Republic of Korea

**Keywords:** Epidemiology, Complex networks, Viral infection

## Abstract

This study estimates the COVID-19 infection network from actual data and draws on implications for policy and research. Using contact tracing information of 3283 confirmed patients in Seoul metropolitan areas from January 20, 2020 to July 19, 2020, this study created an infection network and analyzed its structural characteristics. The main results are as follows: (i) out-degrees follow an extremely positively skewed distribution; (ii) removing the top nodes on the out-degree significantly decreases the size of the infection network, and (iii) the indicators that express the infectious power of the network change according to governmental measures. Efforts to collect network data and analyze network structures are urgently required for the efficiency of governmental responses to COVID-19. Implications for better use of a metric such as R0 to estimate infection spread are also discussed.

## Introduction

The spread of infectious diseases is determined by two factors: the physical and chemical characteristics of the virus, and the social network that defines the structure of contact among people. Humans are not just hosts for viruses. They are actively involved in social contact with others and, as a result, spread the viruses to not just anyone but more or less socially predictable subjects. How humans form social networks affects the overall state and structure of the spread of infection. The current article focuses on this second aspect, that is, social networks. We measure the out-degree distribution of the COVID-19 transmission network in South Korea and predict its function, which allowed us to examine the implications of transmission network information in terms of policy responses to COVID-19, and provides a better informed interpretation of the various reproduction numbers.

Although it is widely known that the characteristics of virus transmission networks are an important determinant of the size and condition of outbreaks, such evidence has been highly limited in policy-making and research on COVID-19, making it challenging to implement evidence-based policies for transmission networks. Social distancing is one such example. Despite the fact that there are local structures for the entire transmission network that disproportionately contribute to the spread of infection, social distancing policies recommend or enforce decreased contact among all members of society. Because this recommendation is not based on scientific analysis of the spread network, the policy may be considered less than efficient or effective.

This lack of scientific examination of transmission networks also limits the interpretation of major indicators of infection, which may lead to inefficient and/or ineffective policies. One important example is the calculation and interpretation of various reproduction numbers (e.g., R, R0). R is an indicator which governmental authorities heavily rely on to determine the current state and future risks of viral infection transmission for quarantine and isolation. However, an estimation process of this indicator suggests that it does not evaluate structural characteristics of the viral transmission network, such as skewness of contact opportunities, thus leading to occasional failures in providing reliable information, which is important for decision-making on resource distribution to control the outbreak.

R is the product of the transmission rate (infection-producing contacts per unit time) and infectious period. To obtain each item, for example, a transmission rate, we assume a model for the infection process (e.g., SIR, SEIHR) and estimate the model's parameters using the number of people at each stage and the rate at which that number increases and decreases. The transmission rate is one of these estimated model parameters^1^. This type of estimation assumes that the transmission rate is determined by the proportion of people at each stage, its rate of increase and decrease, and the rest of the parameters (isolation rate, recovery rate, etc.), which overlook the impact of the transmission network structure. However, the transmission network structure can affect the transmission rates. Even if the proportion of people in each stage and the other parameters are the same, the transmission rate may vary depending on the infection network structure to which people belong.

Meyer et al.'s research is a good example of the impact of network structure. Meyer et al. pointed out that outbreaks under the same R0 (basic reproduction number) can be very different depending on the distribution of out-degrees, the latter meaning the number of transmissions made by each infected person^2^. They compared the power-law distribution, the extreme right-skewed distribution often observed in network measures, to a more moderate Poisson distribution. Though they are expressed by the same R0, the outbreak becomes much more serious if the out-degree follows a Poisson distribution. In contrast, the outbreak may not be as serious if the out-degree follows a power-law distribution because it is likely that a very small number of super-spreaders lead us to overestimate patient increasing rate and R0. By the same logic, the same R0 under a Poisson distribution might suggest that the virus has spread more evenly to the overall population. This result implies that the interpretation of R0, without considering the characteristics of transmission networks, might be incomplete.

The effect of transmission networks on the spread of infection is only theoretically acknowledged, while scientific evidence is absent from current COVID-19 policies. We are not the first to point this out. Existing research have raised the same concerns. However, most previous studies did not address real-world transmission networks between humans. Most existing research deal with networks on social media (e.g., Twitter)^3–6^ or utilize virtual network data^2, 7^. While there are a small number of studies that use real-world network data, they deal with connections between genomes^8^ or contain institutions or organizations in the transmission network's nodes set^9^. That is, technically speaking, they cannot be seen as a network that can demonstrate the transmission dynamics between people. These limitations are understandable given the scarcity of empirical transmission networks between humans. However, we strongly recommend collecting and analyzing empirical network data among humans if we want COVID-19 policies to closely reflect the realities and feasibility between cost and benefit. Therefore, we collected and analyzed real-world transmission network data from people in Korea.

The case of South Korea provides an interesting avenue for this research for two reasons. First, transmission data between humans exists in South Korea. South Korea is famous for active contact tracing^10^. The Infectious Disease Control and Prevention Act (in Korean, 감염병의 예방 및 관리에 관한 법률) in Korea mandates the disclosure of information about confirmed patients, including "movement paths, transportation means, medical treatment institutions, and contacts of patients with the infectious disease"^11^. In compliance with this law, information on infection routes are consistently collected from the initial stage and publicly disclosed on local government websites. By collecting and combining these pieces of information, we can construct the whole network data among humans for the spread of COVID-19 in South Korea. It is a rare opportunity worldwide and is also the first reason we decided to use Korean data.

Second, South Korea has thus far controlled the spread of COVID-19 without relying on strict lockdown policies such as stay-at-home orders or mobility restrictions nationwide. This allowed us to evaluate the structural characteristics and their impact on transmission, while it is less distorted by policy measures that affect naturally existing social ties. Therefore, an analysis of South Korea's infection network is a good reference point for many countries that want effective and efficient epidemic policy through moderate control. We derived several network indicators and their distributions for real transmission data from South Korea, thereby seeking possibilities for improving the efficiency and efficacy of the current policies to curb the COVID-19 outbreak.

More specifically, we attempt to answer the following research questions:

i. What are the characteristics of the COVID-19 transmission network in South Korea?
ii. What are the implications of the distribution of the COVID-19 transmission network index in South Korea from policy and research perspectives?

## Materials and methods

### Data

Our data provide information on those infected and the infection route of each patient made publicly accessible by the Seoul, Gyeonggi-do, and Incheon local governments in South Korea. Because these three municipalities comprise the Seoul metropolitan area, our data show the situation in the capital region of South Korea. Most of the COVID-19 infections in South Korea occurred in the Seoul metropolitan area, Daegu, and Gyeongsanbuk-do. However, since infections in Daegu and Gyeongsangbuk-do occurred rapidly in the early period (February and March), and the government was not well prepared for data gathering, there is a lack of data on the regions' infection routes.

Although webpages containing publicly accessible information differ across local governments, the items commonly disclosed are the confirmation identification number, infection routes, date of confirmation for COVID-19 positivity, and the hospital where the infected person is being treated.

We paid particular attention to the infection route, which comprises a record of key contacts in the infection process identified by the local government and health authorities. This record contains both people and events. If a patient number is recorded as an infection route for a particular patient, the most perfectly specified is the source of infection. However, if a mass infection occurs in a confined space or a person has returned from a foreign country and is found to be a patient, it is difficult to know who infected whom. In this case, the name of the event or place is recorded instead of the patient number. In other words, the record of the infection route is data that allow us to build infection networks, at least in a limited form.

The specific data collection process is as follows: We created a scraping program that automatically collects relevant information from three local government websites. Because information is presented across many pages, it is difficult for human researchers to collect information individually. After the data were collected using this program, we converted the raw data into structured network data. First, we extracted the link information and formed a network of infections between individuals. Individuals are nodes, and links are the infection relationships between them. If another patient is identified in the infection path of one patient, a connection between them is assumed. Simultaneously, two properties of all nodes were extracted and recorded: the confirmed date of each patient and the category of the infection path. Infection path categories describe whether an individual patient's path to infection falls under <Personal>, <Group>, <Overseas>, or <Unknown> . In many cases, events or groups are listed on the infection path information page of individual patients. For example, "Patient No. 2000 was infected via a mass infection in Itaewon" and was recorded on the local government's homepage. In this case, the link information cannot be identified because no interpersonal infection information exists. This person's infection path is categorized into <Group>. <Personal> means that a specific patient infected the patient, <Overseas> means a person was infected from abroad, and <Unknown> is a case where the infection route is unknown.

Finally, our infection network data consisted of patients in the Seoul metropolitan area from January 20, 2020 to July 19, 2020. The network consists of 3,283 nodes and 1,005 links. Links have direction because infections have direction. The frequency of the node infection path category is as follows: <Personal> : 972, <Group> : 869, <Overseas> : 748, <Unknown> : 694.

### Method

We applied three main methods of analysis: network analysis, hypothesis tests on the distributions of network indicators, and virtual structural changes in the network.

**Figure 1:**
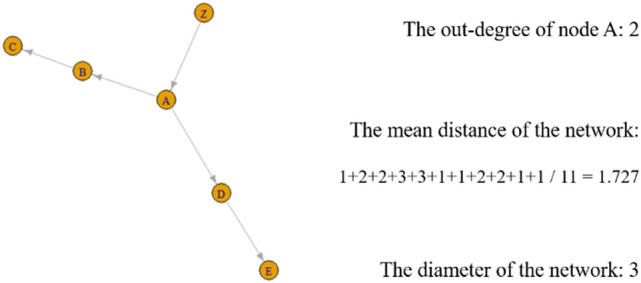
A small directed network.

First, network analysis refers to calculating various network indicators to obtain previously overlooked structural information. Particular attention was paid to the out-degree of each node that constitutes the Korean COVID-19 infection network, mean distance of the network, and diameter of the network. The key to managing infectious diseases is to reduce people's contagion power. From the perspective of network science, this information is expressed through three indicators. One is the out-degree of each node, which means how many direct infections the node has produced. The second is the mean distance of a network, which refers to the average path length between all connected node pairs in the network. That is, we considered only the existing paths and averaged them. The infection network has a tree structure, so the average distance of the network can be described as the average tree depth. We can interpret the mean distance or average tree depth of an infection network as the average potential range of infection. The third is the diameter, which is the length of the longest geodesic in a network. We only considered the distances between connected node pairs. This diameter can also be said to be the maximum tree depth. In the context of infectious diseases, diameter or maximum tree depth shows the most extended "nth transmission" in the network. See Fig. 1 for the intuitive meaning of each indicator. We tried to measure the infectivity of the nodes and the infection network using these three indicators. In particular, the mean distance and diameter are indicators based on the entire network. By analyzing how the two indicators change, depending on time and policy, we identified what changes in the whole network structure are observed depending on time and policy implementation.

Second, we conducted several hypothesis tests on the out-degree distribution to determine the features of the distribution. If an out-degree can be a way of measuring the infectious power of nodes, the features of the distributions of out-degrees are also important. That is, health policies should be determined based on the characteristics of the distribution. As Meyer et al. point out, the infection status of a society can differ depending on the out-degree distribution^2^. Beyond infection networks, network science has long pointed out that the distribution of degree in many networks contains special features. The discussion and debate on scale-free networks and power law initiated by Barabasi is representative^12, 13^. If the out-degree follows the power law, counting on the central tendency, such as the mean of out-degree, which results in rethinking many health policies based on the average trend, is not helpful.

We tested whether the out-degree in the COVID-19 network of South Korea follows the power law. To this end, we followed the procedure proposed by Clauset et al.^14^. First, we estimated the parameters of the power-law distribution, assuming that the out-degrees of nodes are based on the power-law distribution. Then, using bootstrap, we calculated the distances between the 3000 sets of data generated from the estimated distribution and the distribution itself. The 3000 distance values represent random fluctuations that the data would show when they follow the power-law distribution. Then, we compared the distances with the distance between our actual data and the estimated power-law distribution. This determined how many times the distances based on simulated data are farther than the distance between our real data and the estimated distribution. Using the results, we analyzed whether the null hypothesis that the out-degrees of nodes follow the power law should be rejected. The Kolmogorov–Smirnov statistic was used to calculate the distance. Finally, the explanatory power of the power-law distribution was compared with other distributions, which can be an alternative model for fitting a heavy-tailed distribution.

Third, the virtual structural changes in the network were used to estimate the expected effects of network-based health policies. If the health authorities had perfect network information, they would have controlled the most infectious node first in the overall infection network. If successful, it would have had the effect of isolating and eliminating the nodes in the measured infection network. We observed how the overall structure of the infection network and related indicators changed by removing the top nodes on the out-degree. This gave us an idea of the expected effect of health policies using network information.

Additional details are given below. Suppose we remove the nodes whose out-degree is 21 or above. In this case, a certain number of nodes are removed first. Then, we further removed the transmitted nodes from this node as well. We also removed the transmitted nodes from the additionally removed nodes. We repeated this process until there were no more nodes to be removed. This method allowed us to obtain the number of nodes that disappear from the network when we remove the nodes whose out-degree is equal to or larger than the threshold. This process was repeated by changing the out-degree threshold from 51 (maximum value) to 1 (minimum value except 0). We measured the changes in various network indicators as well as the number of nodes removed.

All the analyses described above were performed using R^15^ and its packages, including the following: "tidyverse"^16^ (for data wrangling and visualization), "igraph"^17^ (for network analysis), "lubridate"^18^ (for handling date), "ggraph"^19^ (for visualization), "slam"^20^ (for data wrangling), and "poweRlaw"^21^ (for analyzing power law distribution).

The Seoul National University Institutional Review Board approved this study (IRB No. E2009/003-001, Results of review: Exemption). All methods were carried out in accordance with relevant guidelines and regulations. Our data were de-identified by the Korean government; therefore, the requirement for informed consent was waived.

## Results

### Power law hypothesis test on the out-degree distribution of a node

We analyzed the characteristics of the out-degree distribution of nodes to identify the features of the COVID-19 infection network in South Korea. The out-degrees were calculated using the link direction^22^.

**Figure 2:**
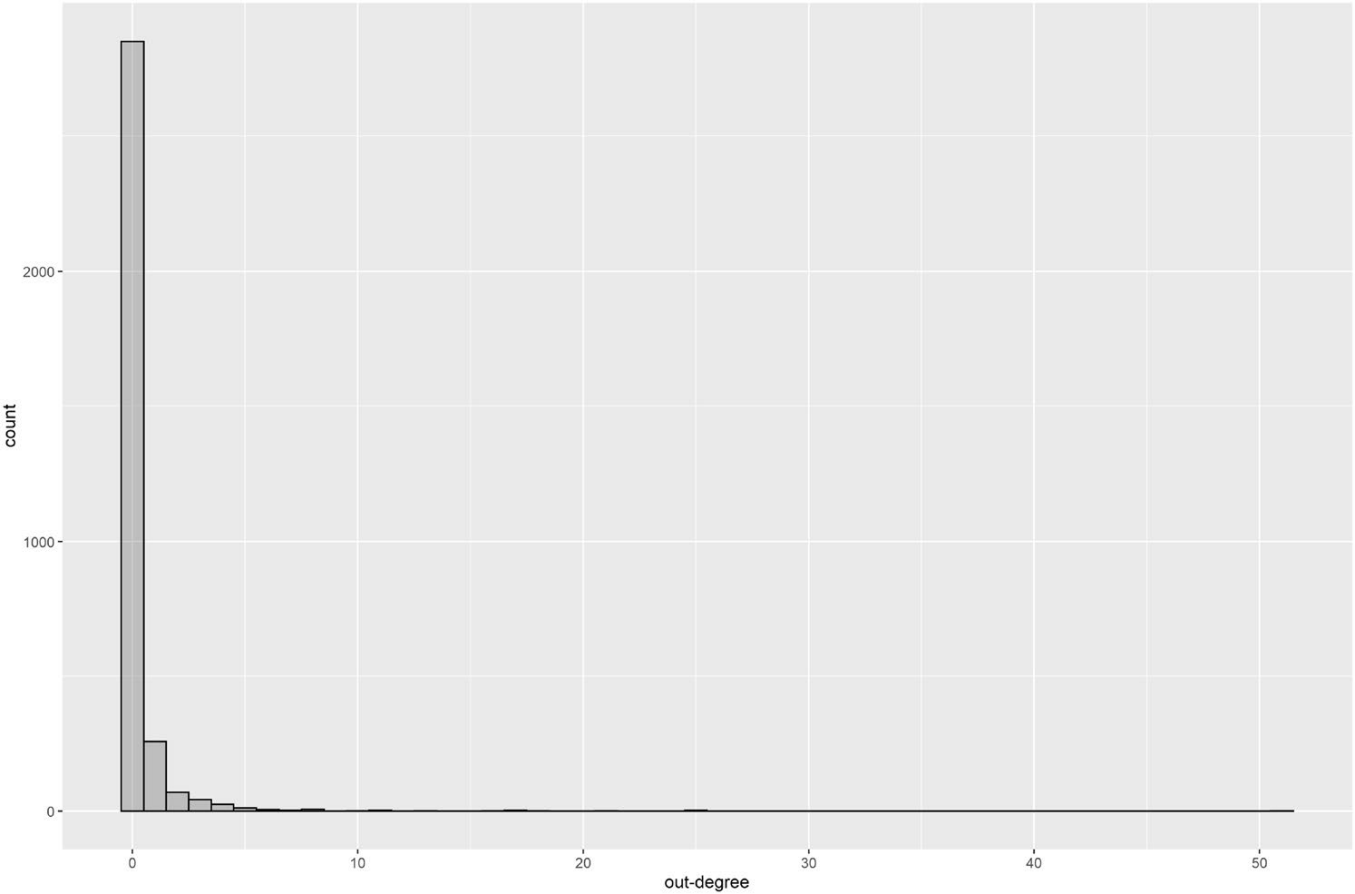
A histogram of out-degree.

First, the out-degree distribution of all nodes is presented in Fig. 2. The pattern of the graph shows a model of distribution with heavy tails.

**Figure 3:**
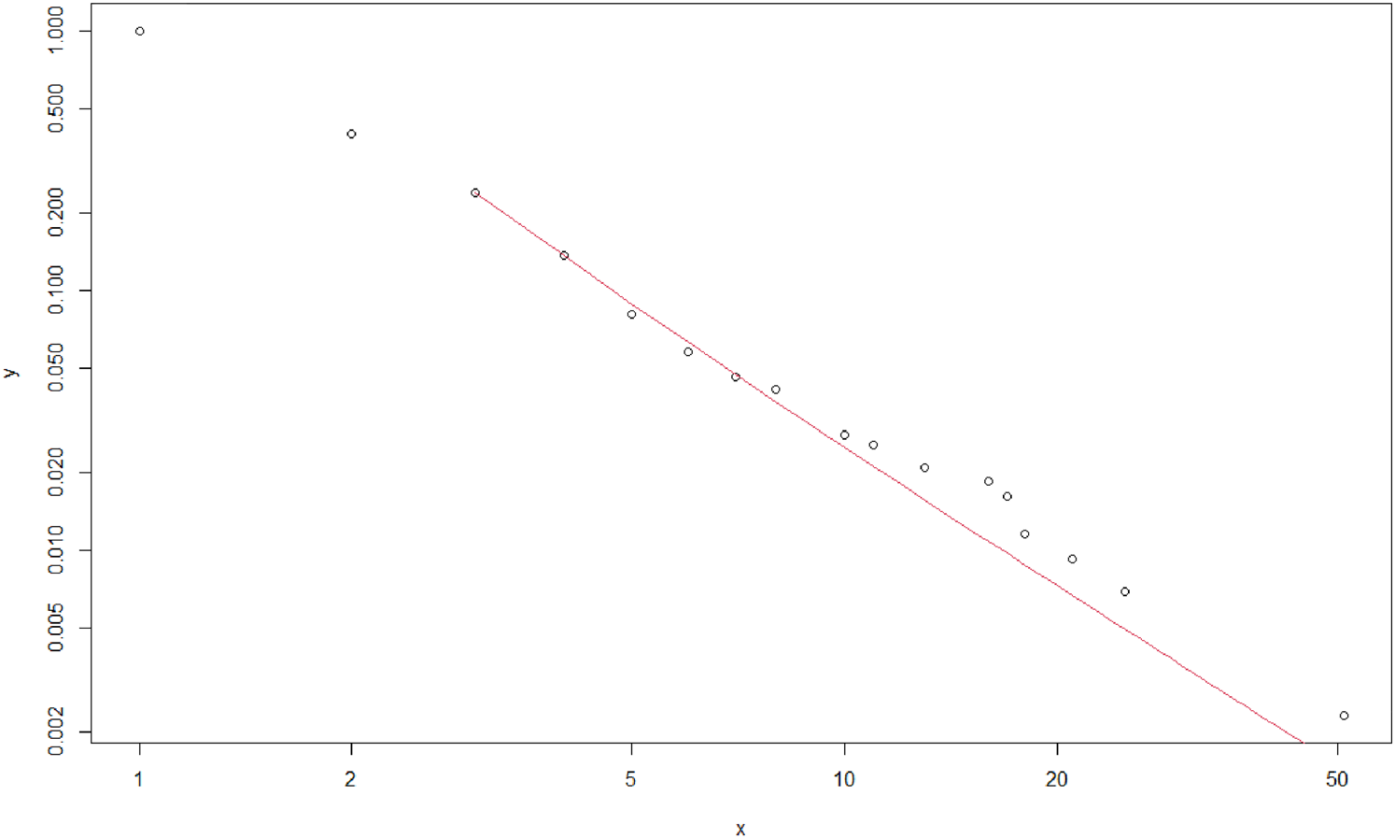
A log–log plot of out-degree. It is plotting the complementary cumulative distribution function of out-degree on doubly logarithmic axes. The red line presents the estimated power-law distribution.

We estimated the parameter of a power-law distribution using the poweRlaw library in R, according to the method proposed by Clauset, assuming our data follow a power-law distribution. As a result, out-degrees with values higher than two were estimated to follow the power-law distribution of the following formula (x: out-degree). Figure 3 presents a log–log plot of out-degrees.

$$p\left(x\right)\propto {x}^{-2.70923}$$

Comparing the distance between the estimated distribution and the 3000 sampled data from the distribution with the distance between the estimated distribution and our actual data, we could not reject the hypothesis that our data follow a power-law distribution. The p-value was 0.610333, meaning that the distances between 61.03% of the sampling data and the estimated model were greater than the distance between our actual data and the model.

Finally, we tested the significance of the distance difference between the three alternative distributions and power-law distribution after estimating the parameters of each distribution based on our data: log-normal distribution, exponential distribution, and Poisson distribution. We used Vuong's likelihood ratio test for this process^23^. The results showed that the power-law distribution has much better explanatory power than the exponential and Poisson distributions. However, the difference in goodness of fit between the power-law and log-normal distribution was indistinguishable. The specific results for each test are shown in Table 1.

**Table 1:** Vuong's likelihood ratio test.

	p-value
Power-law vs log-normal	0.6398939
Power-law vs exponential	0.006273197
Power-law vs Poisson	0.009079904

### Network structure change depending on time and policy

For our research period, the Korean government took three slightly different policy packages to fight the spread of the virus: (i) early stage (01/20/2020–03/21/2020); (ii) social distancing stage (03/22/2020–05/05/2020); and (iii) distancing in daily life stage (05/06/2020–7/19/2020). In the first stage, the Korean government, like any other government elsewhere, did not have a precise policy set. It tried to find the infected as quickly as possible and shut down further spread. As the government's experiences were being accumulated, it came up with the second stage policy named 'social distancing.' This policy set had been effective in constraining the further spread of the virus, which gave the Korean government a room where it could more or less ease the regulations, i.e., the third stage named 'distancing in daily life.'

A summary of each stage's policy is as follows: The Korean government has not ordered strict lockdown policies such as stay-at-home orders or mobility restrictions nationwide. During the "social distancing stage," the Korean central government and local government restricted public institutions' operation. The majority of public institutions such as schools and public libraries were closed. They also recommended that private facilities with conditions prone to infection because of crowded, closed, and confined environments (e.g., indoor sports facilities, religious facilities, entertainment facilities, and private education institutes) be suspended or restrained. The Korean government recommended individuals to take preventive behavior, including refraining from going out, staying home for 3 or 4 days when feeling unwell, keeping a distance of two arms' length from others, washing hands, and ventilating at least twice a day. "Distancing in daily life stage" results from the Korean government's easing of social distancing guidelines. The government has changed the stringency level of policy intervention based on viral transmission data. In the period of "distancing in daily life stage," some public institutions and private facilities resumed operations.

We measured four indicators for each stage. Two of them are the mean distance (average tree depth) and diameter (maximum tree depth), which are explained in the methods section. The other two are the number of human nodes and the number of links. The first is the number of confirmed patients that make up the infection network at that time, and the latter is the number of infections between people that occurred at that time. The results are presented in Table 2.

**Table 2:** Network indicators by period.

Period	Number of human nodes	Number of links	Mean distance of the network	Diameter
Early-stage	691	250	1.447300771	6
Social distancing stage	718	133	1.204968944	3
Distancing in daily life stage	1856	520	1.465359477	5

It is noteworthy that the mean distance and diameter decreased significantly during social distancing.

### The effect of deleting crucial nodes

**Figure 4:**
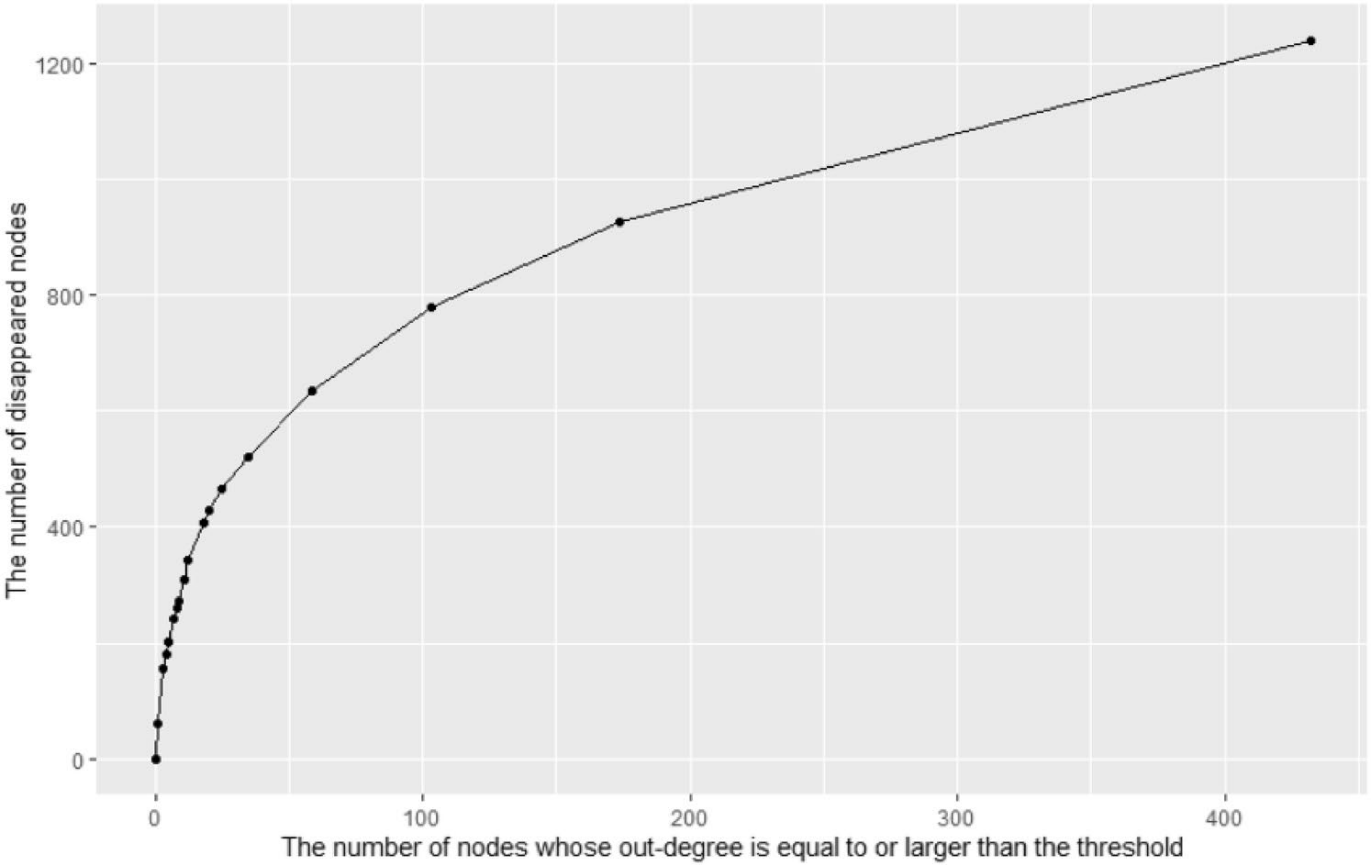
The change in the disappeared nodes' number as we remove the nodes whose out-degree is equal to or larger than the threshold.

**Figure 5:**
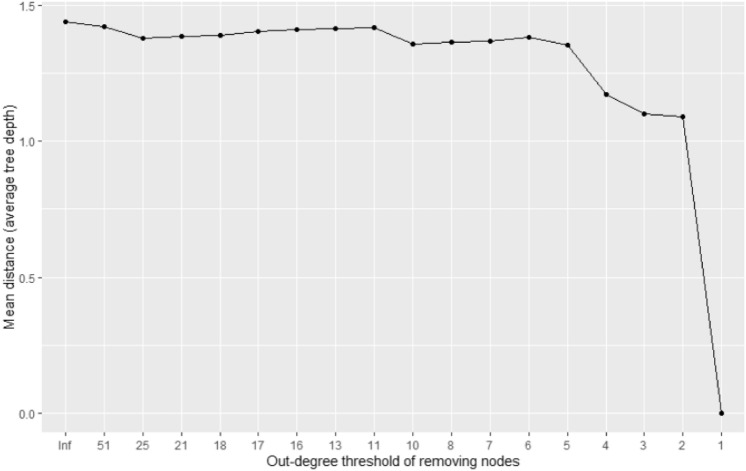
The change in the network's mean distance as we remove the nodes whose out-degree is equal to or larger than the threshold.

**Figure 6:**
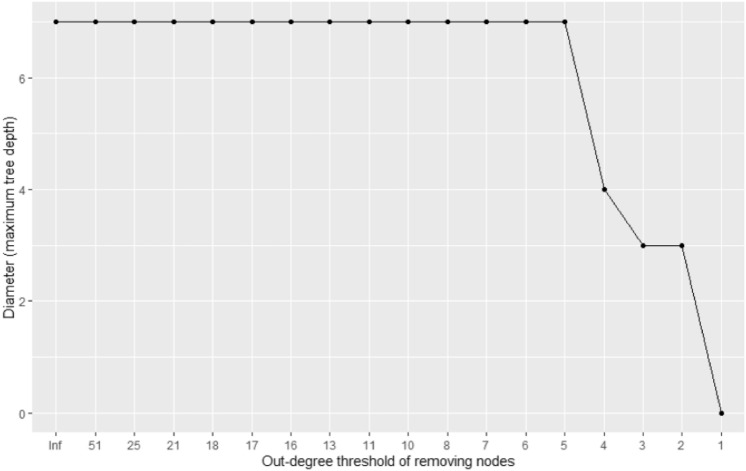
The change in the network's diameter as we remove the nodes whose out-degree is equal to or larger than the threshold.

We analyzed how the overall network structure and key indicators change when important nodes are deleted to anticipate the effects of policies utilizing network information. To this end, we analyzed the resulting structural changes in the network by removing the nodes whose out-degree is equal to or larger than the threshold. We changed the out-degree threshold from 51 (maximum value) to 1 (minimum value except 0) and observed the resulting structure. Since there are 17 values between the maximum and minimum out-degree values (51, 25, 21, 18, 17, 16, 13, 11, 10, 8, 7, 6, 5, 4, 3, 2, 1), we performed 17 virtual node eliminations. In each experiment, we measured the number of nodes eliminated, the mean distance of the network, and the network's diameter. The results are presented in Figs. 4, 5, and 6 and are summarized in Table 3.

**Table 3:** The structure of the network after removing nodes based on out-degree.

The threshold of out-degree	The number of nodes equal to or larger than the threshold	The number of nodes disappeared	Mean distance or average tree depth	The diameter or maximum tree depth
Infinity	0	0	1.43962	7
51	1	60	1.423477	7
25	3	157	1.379068	7
21	4	180	1.385996	7
18	5	201	1.391384	7
17	7	242	1.403056	7
16	8	259	1.409311	7
13	9	273	1.414201	7
11	11	309	1.418219	7
10	12	343	1.357714	7
8	18	406	1.365915	7
7	20	428	1.36965	7
6	25	467	1.382514	7
5	35	522	1.355243	7
4	59	635	1.172949	4
3	103	778	1.102389	3
2	174	926	1.089888	3
1	432	1239	0	0

Figure 4 shows that the larger the out-degree threshold, the more efficiently nodes disappear from the network. In other words, when we remove the nodes with a large threshold, even though the number of nodes removed first is relatively small, the number of nodes disappearing from the network in the final is large. For example, Table 3 shows that 201 nodes disappeared when we remove the five nodes whose out-degree is equal to or larger than 18, but only 467 nodes disappear when we remove the 25 nodes whose out-degree is equal to or larger than 6, not 1005 (201*5) nodes. This phenomenon shows that the intervention's effect of removing high out-degree nodes can be large and efficient.

Figures 5 and 6 show that the network distance is relatively robust to eliminating nodes with a high degree. They are maintained or slightly changed until we lower the threshold to five. This is estimated to be due to nodes with relatively low out-degree but construct connections with relatively long path distances. According to Fig. 6, the diameter or maximum tree depth is maintained at seven until we lower the threshold to five, which indicates that the connection between nodes with the longest path distance does not necessarily contain the node with the high out-degree.

**Figure 7:**
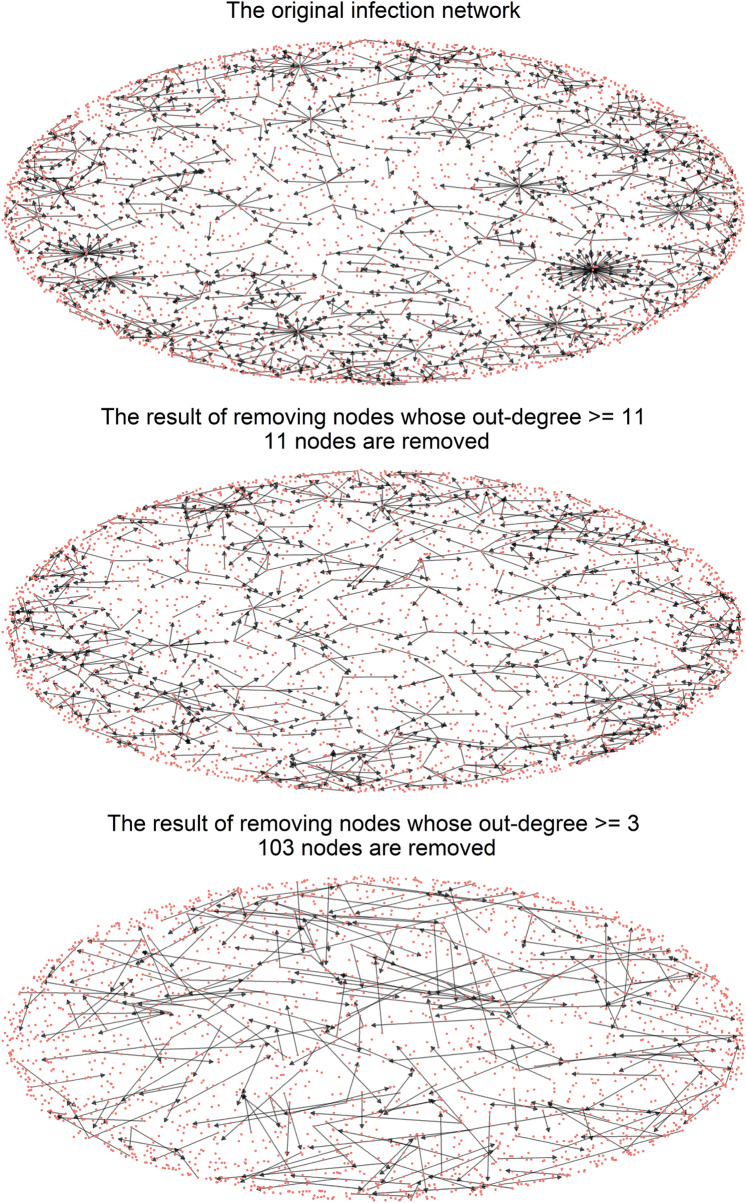
The change in the network's shape as top nodes in the out-degree are removed.

Figure 7 depicts the network's shape when the top nodes based on out-degree are removed, changing the threshold. We visualized three networks using the Kamada-Kawai layout. The first network is the original infection network, which consists of 3,283 nodes. The second network is the result of removing nodes whose out-degree is equal to or larger than 11. In this case, we removed the top 11 nodes on the out-degree. The third network is the result of eliminating nodes whose out-degree is equal to or larger than three. In his case, we removed the top 103 nodes on the out-degree.

## Discussion

The results of our study are summarized as follows. First, the distribution of out-degrees follows an extremely positive skewed distribution. Second, removing the top nodes of the out-degrees efficiently reduces the number of patients in the network. Third, existing policies have a changing effect on the infection network structure. During the social distancing period, the mean distance and diameter of the infection network were significantly reduced.

This study has four main implications. First, it indicates the importance of interpreting network indicators to analyze the key infection indicators currently utilized. For example, various reproducibility indices (R) do not take into account network structural effects. However, the network structure can affect the rate of increase in the number of infected persons or the transmission rate. If the distribution of out-degrees is an extremely positive skewed distribution, as we have revealed in the actual infection network data in Korea, the basic reproduction index may overestimate the actual risks. Therefore, considering various network and infection indicators together allows for evidence-based decision making during COVID-19 policy formulation processes.

Second, the study suggests that by analyzing various network indicators and their distributions, quarantine authorities can make their policies more feasible. If the degree distribution of infection networks is an extremely positive skewed distribution, as our current data show, screening and managing nodes with high infection potential may be more efficient than interventions targeting the entire population. This is evidenced by the significant effect of the virtual deletion of the top nodes based on out-degree from our data. Conversely, if the distribution of network indicators is close to a normal distribution, comprehensive policies targeting the entire population may be useful. Furthermore, this study demonstrates how the various network indicators related to infectious forces vary depending on the significant policies already in place. This means that network indicators can supplement existing indicators in measuring the effectiveness of policies.

Third, our study suggests that contact tracing is as important as testing for COVID-19 infection and that we need to invest more resources. Our data, summarized in the <Data> subsection, indicate that the infection path investigation is relatively insufficient compared to the infection itself. The <Unknown> or <Group> categories account for a vast proportion. However, as we have seen, one neglected patient can produce a massive number of patients. The positively skewed out-degree distribution proves this. In order to curb the number of patients in the right tail, it is necessary to track the infected person's contact path as much as possible. In short, it is as necessary to invest resources to improve the contact tracing capacity as it is to invest in the ability to check for infections.

Fourth, our results show that nodes with high out-degree and nodes consisting of long-distance connections can be different. In virtual deletion of nodes based on out-degree, we observed that the network distance is relatively robust to eliminating nodes with a high out-degree. It is already well known that finding super-spreaders is essential for managing infectious diseases. However, infection connections in long path distances are also important. Suppose a long-length infection network consisting of nodes that are not high in the out-degree exists, and the government only pays attention to controlling the nodes with a high out-degree. In that case, this can lead to additional infections between groups or people geographically or conceptually far from each other. Infection connections with this potential do not necessarily have to be made up of nodes with a high out-degree. It informs us that measurements on different indicators are needed to cope with different threats. However, we cannot determine whether this is a universal phenomenon because we did not analyze other infection networks. Furthermore, a closer analysis centered on distance indicators is needed. However, this is not a central topic in this paper, so we left it as a research topic to be carried out in the future.

This study has some limitations. Above all are the limitations to the data. The data we used are missing information from Daegu and Gyeongsangbuk-do, which have produced many patients. In addition, our data, based on government announcements, inevitably underestimate links between individuals. For example, suppose a patient is infected from a group infection. In that case, it can be assumed that a specific individual existed in the patient's infection path in reality. However, the data did not state this other individual who infected the patient.

However, perfect data cannot be obtained. Furthermore, if data from Daegu and Gyeongsangbuk-do are secured, and the infection path data, including the interpersonal infection network, is complete, it will likely support the conclusion of this study more strongly by expanding the interpersonal link much more than it is now. This is because group infections are likely to include super-spreaders, that is, high out-degree nodes. It is assumed that significant events in Daegu will also be the same. In sum, this study utilized actual data to provide limited but meaningful results.

## Data Availability

The data are publicly available at https://www.seoul.go.kr/coronaV/coronaStatus.do (Seoul), https://www.gg.go.kr/bbs/board.do?bsIdx=722&menuId=2903#page=1 (Gyeonggi), https://www.incheon.go.kr/health/HE020409 (Incheon).
